# Bibliographical Notices

**Published:** 1855-10

**Authors:** 


### EDITORIAL DEPARTMENT.

### BIBLIOGRAPHICAL NOTICES.

*Outline of Chemical Analysis.* Prepared for the Chemical Laboratory at Gieseen. By Dr. Heinrich Will. Translated by Drs. Breed and Steiner. Boston and Cambridge: James Munroe & Co., 1855.

The first German edition of this work, came out under the sanction of Liebig, its author having for many years assisted that eminent chemist and won an enviable reputation for himself. It has, therefore, the double advantage of being the production of one who has mastered the difficulties of his art, and of a teacher who has guided many to a knowledge of practical analytical chemistry. Its compass is small but it contains much valuable information and is well adapted to the purpose for which it was intended, an introduction to the art of analytical chemistry.

It possesses the merit of not confusing the student by too great a number of tests for each substance he is called upon to examine. A few characteristic reactions are given, the rest are entirely neglected.

The order in which the substances are arranged differs in no important particular, from those commonly adopted in works of this character. In two respects, however, we prefer Fresenius. He first instructs the student to make and test his own reagents and thus gives him a confidence in his future operations he could not otherwise have. Such a mode of instruction is peculiarly necessary in our country on account of the extreme carelessness with which chemicals are made. It is exceedingly difficult to procure any reagent perfectly pure. The other advantage possessed by Fresenius' book is the greater attention paid to the use of the blow-pipe. The preliminary remarks on this subject in the book before us, and indeed the whole chapter on manipulations, are too meagre to be of any practical benefit to the pupil.

The examples for practice in quantitative analysis are very judiciously selected, and the description of the processes, though brief, is clear and satisfactory. The tables of reactions at the end, will be found very useful.

*A Manual of Clinical Medicine and Physical Diagnosis.* By T. H. Tanner, M. D. To which is added, the Code of Ethics of the American Medical Association. Philadelphia: Blanchard & Lea, 1855.

The increased attention paid of late years to the study of clinical medicine has produced a great number of text-books on the subject. Manuals of all kinds have become very abundant, while clinical lectures and works on the principles of medicine and surgery have been multiplied beyond all precedent. The students of this generation ought to be far ahead of their predecessors, for never before has so much pains been bestowed upon medical education or the facilities for acquiring it been so liberally furnished.

The little book before us is an admirable guide to the examination of the sick man. Its directions are brief, simple and practical, and yet sufficiently explicit for the purposes of the student or the young practitioner.

The plan upon which it is constructed is also good. After some preliminary remarks on the facility of observation and the general conduct of the medical practitioner, the author proceeds to lay down rules for the clinical examination of a patient. If these be properly followed, they will be found quite sufficient to secure a full knowledge of the case in question. Then we have formulæ for taking notes of cases, for conducting post mortem examinations and recording their results, for investigating the diseases of the insane, for examining persons desiring to have their lives insured and for making medico-legal inquiries. Then follow descriptions of the various instruments used in the investigation of disease, the microscope, the opthalmascope, the spirimeter, the test-tray, &c. Disease is next considered in its nature, its causes, its classification, diagnosis, prognosis and terminations. We then have a chapter on the various circumstances which modify disease, age, sex, hereditary tendency, temperament, diathesis, habit, climate and temperature. Symptomatology comes next in order, and the different symptoms are carefully classified under appropriate heads, according to the systems and organs in which they develop themselves. The diagnosis of natural from feigned disease forms the theme of another chapter, and a table of feigned diseases and their detection is given. Physical diagnosis is then treated of, after which a special chapter is appropriated to the diagnosis of thoracic diseases, and another to that of diseases of the skin. Willan's classification, as modified by Biett, is adopted by the author. The parasites are next considered, and lastly, the chemical and microscopical examination of blood, expectorated matter, the rejected contents of the stomach and the urine.

From this brief summary, it will be seen that the little book under consideration covers much ground ; wide, however, as is its field, it is by no means slight and superficial. It is evidently the production of an acute and sagacious observer, who has met and vanquished difficulties, and we are sure that no one can read it without both pleasure and profit.

*An Essay to Prove the Contagious Character of Malignant Cholera; with Brief Instructions for its Prevention and Cure.* By Bernard M. Byrne, M. D., Surgeon U. S. Army. Second edition, with additional notes by the author. Philadelphia: Childs & Peterson, 1855.

Dr. Byrne confounds contagion with communicability, a distinction which has been lately made with special reference to explaining the phenomena of epidemics. The communicability of cholera by human intercourse we think he has conclusively established, and indeed it is an opinion which is daily gaining ground. Those who wish to study the arguments of the advocates of the communicability of cholera will do well to read attentively this volume of Dr. Byrne.

*Letters to a Young Physician just Entering upon Practice.* By James Jackson, M. D., LL. D. Boston : Phillips, Simpson & Co., 1855.

Near the close of a long and active life, the Emeritus professor of theory and practice of physic in the University at Cambridge, comes forward to counsel and instruct the young in the more important of their daily du ties. He writes with that sort of fatherly interest which men who are long engaged in professional teaching are apt to acquire. His aim has been to communicate as far as practicable his own experience to his readers, and to furnish them with directions for their future even from his past. Many interesting questions are discussed, and some uncommon affections described, and ordinary diseases studied from a new point of view. We heartily commend the book to all youthful practitioners of the healing art.

*The Diseases of the Human Teeth.*—Their Natural History and Structure, with the mode of Applying Artificial Teeth, etc., etc. By Joseph Fox, Surgeon Dentist; Lecturer on the Structure and Diseases of the Human Teeth at Guy's Hospital, etc., etc.; and Chapin A. Harris, M. D., D. D. S., etc., etc.; with two hundred and fifty illustrations. Imp. 8vo. pp. 440; Lindsay & Blakiston: Philadelphia, 1855.

We have received from the publishers, Messrs. Lindsay and Blakiston, a copy of the above beautifully gotten up work, and we do not know that we can give a better idea of the book than by copying the following from the preface of the American edition.

The first complete edition of this work was published in 1806; and notwithstanding the rapid progress which dental surgery has made subsequently to this period, it still occupies a high place in the literature of this department of medicine. It has been more extensively quoted than any other treatise upon the same subject, and has passed through three editions in England. The last of which was published in 1833. There is still, both in Europe and America, a steady demand for it.

The author during his professional career enjoyed a high reputation as a practitioner of dental surgery; and for many years previously to his death was a lecturer on this branch of medicine in Guy's Hospital. Although he has passed from among the living, he has left behind him a memorial which will perpetuate his name to the latest period of time. Wherever, and so long as this branch of surgery shall be practiced by educated men, will the name of Fox be held in grateful remembrance.

In preparing the work for republication in this country, the editor has found it necessary to make numerous and extensive additions, equal to three-fourths of the original text, in order to adapt it to the present state of dental practice. He has also added seven plates, remodeled the arrangement of the subjects, and placed over each an appropriate caption.

The whole of the original text, with the exception of about twelve pages will be found in the present edition of the work. The scope of the work however has not been materially enlarged—the aim of the editor having been to supply the details of the subsequent improvements in practice.

